# The 25th Anniversary of the American Society of Clinical Investigation’s Korsmeyer Award

**DOI:** 10.1172/JCI168855

**Published:** 2023-02-15

**Authors:** Benjamin D. Humphreys

**Affiliations:** Division of Nephrology, Department of Medicine and Department of Developmental Biology, Washington University in St. Louis, St. Louis, Missouri, USA.

The American Society of Clinical Investigation (ASCI) presents the Stanley J. Korsmeyer Award to one or two scientists who have made substantial contributions to our understanding of disease and to mentoring the next generation of physician-scientists each year. Originally named the ASCI Award, it was renamed in 2006 to honor the first recipient of the prize, Dr. Stanley J. Korsmeyer (Figure 1), a physician-scientist respected widely for both his impactful science and his own remarkable legacy of mentorship. To recognize the 25th Anniversary of the Korsmeyer Award, the Journal of Clinical Investigation is featuring a series of Review and Viewpoint articles from past Korsmeyer Award winners (Table 1), which will be published over the course of 2023.

## Stan Korsmeyer’s personal story

Growing up on a farm in central Illinois, Korsmeyer originally wanted to become a veterinarian. He was the youngest contestant in the history of the Illinois State Fair to show a grand champion pair of Hampshire hogs, at age 14 (1). While an undergraduate at the University of Illinois Urbana-Champaign, an early mentor and local veterinarian named Robert Goodin suggested that he pursue a career in medicine instead of veterinary science. Korsmeyer took this wise advice and enrolled at the University of Illinois College of Medicine in Chicago, graduating with his MD in 1976, after publishing a first-author manuscript in the *New England Journal of Medicine* on autoantibodies in Crohn’s disease as a medical student (2).

Following training in internal medicine at the University of California at San Francisco, Korsmeyer moved to the National Cancer Institute in Bethesda as a fellow in molecular oncology in the laboratories of Thomas Waldmann and Philip Leder. This training led to an interest in highly recurrent chromosomal translocations in patients with hematologic malignancies. In the 1980s, Korsmeyer and others defined a translocation between chromosomes 14 and 18 that placed the regulatory elements for the heavy-chain immunoglobulin locus upstream of an uncharacterized gene called BCL2, which itself was not altered and whose function was unknown (3–5). This translocation was found in most follicular lymphomas. Korsmeyer moved his laboratory to Washington University School of Medicine in 1986, after which his group and others showed that BCL2 caused cancer not by driving cell proliferation, but rather by preventing programmed cell death, or apoptosis (6–8). This observation was paradigm shifting, because cancer had previously been thought to be a disease of increased cell proliferation — not dysregulated cell death. He continued to work at the forefront of apoptosis research and discovered many other BCL2 family members, including the prodeath gene BAX (in contrast with the anti–cell death BCL2), BID, and BAD. These advances ultimately also led to the development of a BCL2 inhibitor, Venetoclax, that is now used to treat many hematologic malignancies (9).

Although he was a nonsmoker, Korsmeyer developed lung cancer and ultimately died from it in 2005, at the age of 54. Korsmeyer’s brilliance was recognized during his lifetime. He was elected to the National Academy of Sciences at age 45 as well as to the Institute of Medicine, the American Academy of Arts and Sciences, and the American Philosophical Society. He received the Bristol-Myers Squibb Award, the Stratton Medal from the American Society of Hematology, and the International Award for Cancer Research from the Pezcoller Foundation and the American Association for Cancer Research, among many others. Korsmeyer was recruited to the Dana-Farber Cancer Institute in 1998, where he continued his scientific work, his mentoring, and institutional leadership until his untimely death.

## Korsmeyer’s enduring legacy

Korsmeyer is remembered equally for his personal attributes, especially his generosity of spirit, and his passion for mentorship. Tim Ley, Korsmeyer’s friend and colleague at Washington University has written, “When a graduate student told Stan that he was struggling, Stan smiled and replied, ‘Okay, let’s struggle together,’ and he meant it” (10). Ben Ebert, the 2021 Korsmeyer Award winner, has said of Korsmeyer that “…he is legendary at the Dana-Farber, as a mentor and as a scientist. He had such a profound influence on the entire institution through his leadership, and his former mentees are among the current leaders at the Dana-Farber. For many, he has been the premier role model of a physician-scientist and mentor” (11).

The prestige of the Korsmeyer Award is reflected by the gratitude expressed by prior recipients. Peter Tontonoz, MD, the 2022 Korsmeyer Award awardee, wrote, “If you had asked me, ‘What’s one recognition that would mean a lot to you, other than the Nobel Prize?’ I would have mentioned the Korsmeyer Award” (12). Bill Kaelin, MD, Nobel Laureate, and winner of the 2012 Korsmeyer Award with Gregg Semenza, wrote, “If there was ever a resounding counterexample to ‘nice guys finish last,’ it was Stanley Korsmeyer. Anyone who ever had the privilege of knowing Stan knows that he was one of the nicest people you could ever meet in addition to being an absolutely brilliant scientist. He was (and remains) one of my heroes, and for that reason, I could not be more proud to win this award” (13).

Korsmeyer’s legacy lives on in the form of his highly successful trainees and through the Korsmeyer Award itself. The Korsmeyer Award winners exemplify core values of the ASCI — physician-scientists making exceptional scientific contributions and making a resounding commitment to mentoring the next generation. The ASCI and *Journal of Clinical Investigation* are proud to commemorate the 25th anniversary of the Korsmeyer Award with a collection of articles contributed by past winners.

## Figures and Tables

**Figure 1:**
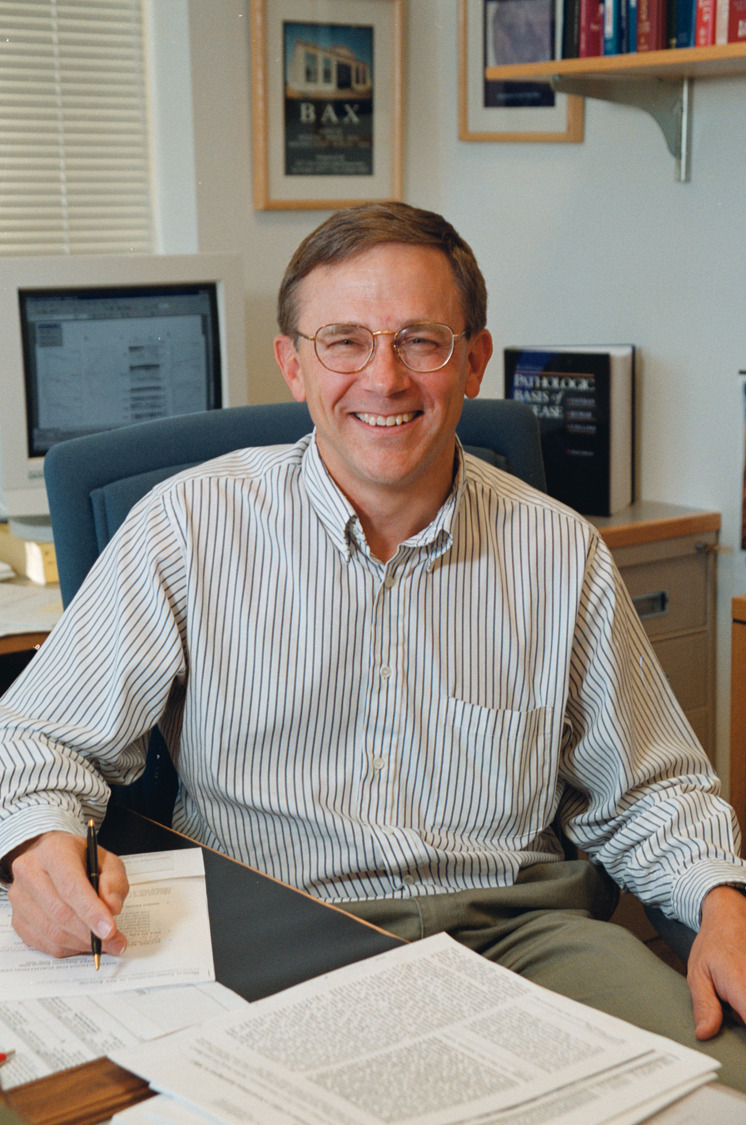
Stanley J. Korsmeyer.

**Table 1:**
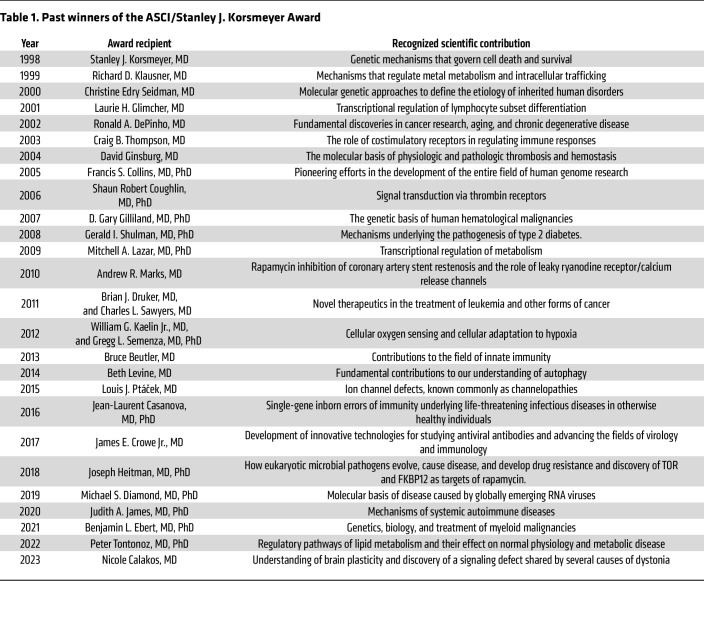
Past winners of the ASCI/Stanley J. Korsmeyer Award

## Version 1 02/15/2023

Electronic publication
